# Evaluating the impact of COVID-19 on vertigo and hearing impairment: A post-recovery analysis

**DOI:** 10.1097/MD.0000000000038819

**Published:** 2024-07-05

**Authors:** Sarah Alshehri, Khalid A. Alahmari

**Affiliations:** a Otology and Neurotology, Department of Surgery, College of Medicine, King Khalid University, Abha, Saudi Arabia; b Medical Rehabilitation Sciences, College of Applied Medical Sciences, King Khalid University, Abha, Saudi Arabia.

**Keywords:** COVID-19, hearing loss, post-acute sequelae, SARS-CoV-2, sensory disorders, vertigo

## Abstract

The COVID-19 pandemic, caused by the novel coronavirus SARS-CoV-2, has manifested with respiratory symptoms and a spectrum of extra-pulmonary complications. Emerging evidence suggests potential impacts on the auditory and vestibular systems, but the extent and nature of these effects in recovered individuals remain unclear. This study aimed to investigate the prevalence and severity of vertigo and hearing impairment in individuals who have recovered from COVID-19 and to identify potential risk factors associated with these sensory symptoms. A cohort of 250 recovered COVID-19 patients was assessed. Standardized questionnaires, including the Dizziness Handicap Inventory and the Vertigo Symptom Scale, were used to evaluate vertigo. Hearing assessment was conducted using pure-tone audiometry, speech audiometry, tympanometry, and oto-acoustic emissions testing. Logistic regression analysis was performed to assess the association between COVID-19 severity and the occurrence of sensory symptoms, controlling for confounding variables such as age and comorbidities. Of the participants, 10% reported vertigo, varying severity. Hearing assessments revealed that most participants had normal hearing, with an average speech discrimination score of 94.6. Logistic regression analysis indicated a significant association between severe COVID-19 and an increased likelihood of vertigo (OR 2.11, 95% CI 1.02–4.35, *P* = .043) and hearing impairment (OR 3.29, 95% CI 1.60–6.78, *P* = .002). This study suggests a significant association between COVID-19 severity and vertigo and hearing impairment prevalence. The findings underscore the importance of sensory symptom assessment in the post-recovery phase of COVID-19, highlighting the need for comprehensive healthcare approaches to manage long-term sequelae.

## 1. Introduction

The advent of COVID-19, induced by the severe acute respiratory syndrome coronavirus 2 (SARS-CoV-2), heralded an unparalleled global health emergency.^[1]^ Commencing in the latter part of 2019, this pandemic has imposed substantial challenges upon healthcare infrastructures, macroeconomic stability, and societal dynamics worldwide.^[2]^ Initially characterized by its primary manifestation as a respiratory illness, the disease has increasingly been recognized for its multifaceted nature, impacting multiple organ systems and presenting a broad spectrum of clinical manifestations.^[3]^

COVID-19’s initial characterization centered on respiratory symptoms, including fever, cough, and dyspnea.^[4]^ These symptoms have been identified as hallmark features of the disease, forming the basis of early diagnostic criteria and public health responses.^[4]^ However, as the pandemic progressed, a more complex picture of the disease began to emerge. Clinicians and researchers worldwide started to report diverse symptoms extending beyond the respiratory system.^[5]^ This included gastrointestinal disturbances, neurological manifestations like anosmia and ageusia, cardiovascular complications, and a range of dermatological presentations.^[6]^ These observations underscored the systemic nature of the infection and its capacity to affect extra-pulmonary organ systems.^[6]^

One area that has garnered significant attention is the potential impact of COVID-19 on the auditory and vestibular systems.^[7,8]^ Reports of auditory symptoms such as tinnitus, hearing loss, and vertigo in patients with COVID-19 have been increasingly documented, sparking scientific interest in understanding the relationship between the virus and these sensory systems.^[9]^ The auditory and vestibular symptoms associated with COVID-19 present a new dimension to the disease’s complexity and pose unique challenges in clinical management.^[10–12]^

Vertigo, frequently characterized as a perceptual phenomenon of rotation or motion, represents a symptomatic manifestation that can markedly impair an individual’s life quality.^[13]^ It can be accompanied by dizziness, nausea, and balance issues, and arises from various causes, including inner ear disorders, neurological conditions, and, as recent evidence suggests, viral infections.^[14]^ The development of vertigo symptoms in the context of COVID-19 has raised critical questions about the mechanisms through which the virus may influence the vestibular system.^[15,16]^ The vestibular system, housed within the inner ear, is integral to balance and spatial orientation.^[17]^ It comprises a complex network of sensory organs that detect head movements and gravitational forces.^[17]^ Viral infections, such as those caused by herpes simplex virus and cytomegalovirus, have previously been implicated in vestibular disorders.^[18]^ This background suggests a plausible pathway for SARS-CoV-2 to impact the vestibular system, either through direct viral invasion or via the immune response it elicits.^[19]^

Similarly, hearing impairment in the context of COVID-19 has raised significant concerns.^[20]^ Hearing loss can manifest as sensorineural, conductive, or mixed types, with sensorineural hearing loss drawing particular attention due to its association with inner ear or auditory nerve damage.^[20]^ The relationship between viral infections and sensorineural hearing loss is well-established, with viruses such as mumps, measles, and rubella known to cause auditory dysfunction through mechanisms including direct viral invasion, immune-mediated damage, or vascular compromise.^[21]^ The occurrence of hearing loss in COVID-19 patients has thus prompted investigations into the virus’s potential to similarly affect auditory function.^[22]^ The auditory system, like its vestibular counterpart, is reliant on specialized structures for its function.^[23]^ The cochlea, for example, plays a critical role in transducing sound vibrations into electrical signals for the brain to process. Disruption of the cochlea’s hair cells or the auditory nerve can lead to significant hearing impairment.^[24]^

This study aims to explore the connection between COVID-19 and 2 specific sensory symptoms: vertigo and hearing impairment. Our investigation is particularly focused on individuals who have recovered from COVID-19, aiming to discern the prevalence and severity of these symptoms in this population. By examining the occurrence and severity of vertigo and hearing impairment in COVID-19 survivors, we aim to contribute to the growing body of knowledge regarding the long-term effects of the virus. Additionally, we seek to identify potential risk factors contributing to these sensory symptoms in COVID-19 survivors, including variables such as viral load, age, and underlying comorbidities.

## 2. Materials and methods

### 2.1. Study design, settings, and ethical considerations

This research employs a cross-sectional observational methodology to explore the correlation between vertigo and auditory deficits in individuals post-recovery from COVID-19. The study was conducted at KKU Hospital and ENT Clinic, a primary care medical center specializing in infectious diseases and audiology, located in Abha, Aseer. The investigation was executed in alignment with the ethical tenets outlined in the Declaration of Helsinki. The ethical sanction was procured from the Institutional Review Board at DRS, KK University, as denoted by the ethical clearance number ECM# 2022-2235. Prior to inclusion in the study, written informed consent was secured from all participants, ensuring compliance with ethical standards.

### 2.2. Participants

Patient enrollment in the study utilized a multi-step process with purposive sampling to ensure a representative cohort of recovered COVID-19 patients. Initially, potential participants were identified from a database of patients who had tested positive for COVID-19. The database was compiled from various healthcare facilities to ensure diversity. Inclusion criteria were adults aged 18 years and older with a confirmed COVID-19 diagnosis via a positive Polymerase Chain Reaction test and at least 4 weeks free from symptoms.^[25]^ Exclusion criteria included preexisting vestibular disorders, non-COVID-19 related hearing impairments, and severe cognitive or communication impairments. Purposive sampling targeted individuals meeting these criteria across different ages, genders, and COVID-19 severity levels. This technique aimed to select participants likely to provide relevant data for the research objectives. Once identified, potential participants were contacted and screened for eligibility. Eligible participants received detailed study information and provided written informed consent before participation.

The study focused on a cohort of 250 adults who had previously contracted COVID-19, adhering to stringent inclusion and exclusion criteria to explore the association between COVID-19 and sensory symptoms. Inclusion criteria were adults aged 18 years and older with a confirmed COVID-19 diagnosis through a positive Polymerase Chain Reaction test, and at least 4 weeks free from symptoms. Exclusion criteria included preexisting vestibular disorders and non-COVID-19-related hearing impairments. Severe cognitive impairment or communication difficulties were also excluded. This careful selection aimed to ensure the validity and reliability of the study’s findings, particularly in understanding the potential long-term sensory impacts of COVID-19.

### 2.3. Sample size estimation

For the sample size estimation of our study, we utilized G*Power statistical software, adhering to standard biomedical research parameters. An alpha level was set at 0.05, to achieve 80% power to detect a moderate effect size, approximately quantified as 0.3, based on preliminary data. This effect size reflects the expected variations in the prevalence of sensory symptoms across different COVID-19 severity levels. Taking these parameters into account, along with an anticipated dropout rate of about 10%, our analysis indicated that a total of 250 participants would be adequate. This sample size effectively balances the necessity for sufficient statistical power to discern significant effects, while also considering the practical aspects of participant recruitment and resource constraints, thus ensuring the study’s feasibility and the validity of its outcomes.

### 2.4. Vertigo assessment

The assessment of vertigo symptoms in our study was meticulously conducted using 2 standardized questionnaires: the Dizziness Handicap Inventory (DHI) and the Vertigo Symptom Scale (VSS).^[26]^ The DHI is designed to evaluate the impact of dizziness and unsteadiness on an individual’s daily life, providing a comprehensive understanding of how these symptoms affect day-to-day activities.^[27]^ Concurrently, the VSS was employed to gauge the frequency and intensity of vertigo symptoms.^[28]^ This dual-assessment approach, leveraging the DHI and VSS, allowed for a nuanced and detailed evaluation of vertigo, capturing both the qualitative aspects of how these symptoms affect patients’ lives and the quantitative aspects of symptom frequency and severity.^[29]^

### 2.5. Hearing assessment

Hearing assessments in our study were comprehensive and conducted using state-of-the-art audiological techniques. The evaluation began in a soundproof booth where pure-tone audiometry was performed, measuring air and bone conduction thresholds across a range of frequencies (250 Hz, 500 Hz, 1000 Hz, 2000 Hz, 4000 Hz, 6000 Hz, and 8000 Hz).^[30]^ Additionally, speech audiometry was employed to assess speech discrimination scores (SDS), providing insights into the participants’ ability to discern speech, which is critical in understanding the practical implications of hearing loss. Tympanometry was another key component of our assessment, offering valuable information about middle ear function, including tympanic membrane mobility and the conduction of sound to the inner ear.^[30]^ Finally, otoacoustic emissions testing was utilized, a sensitive technique that evaluates cochlear function by recording sounds emitted by the inner ear.^[30]^ This comprehensive hearing assessment protocol, encompassing pure-tone and speech audiometry, tympanometry, and otoacoustic emissions testing, provided a multifaceted view of the auditory health of the COVID-19 survivors in our study, crucial for understanding the full extent of the virus’s impact on auditory function.^[30]^

### 2.6. Data analysis

Statistical computations were executed utilizing the Statistical Package for the Social Sciences (Statistics version 24, IBM^®^, USA). Descriptive statistical methods were employed to collate demographic data, clinical attributes, and the incidence rates of vertigo and auditory impairments. Quantitative variables, exemplified by pure-tone audiometry outcomes, were evaluated using mean values and standard deviations. Conversely, qualitative variables were delineated through frequencies and percentage distributions. Logistic regression analysis was applied to determine the correlation between COVID-19 and the incidence of vertigo/auditory impairments. The regression framework incorporated potential confounding factors, including age, gender, and comorbidities. The computation of Odds Ratios (OR) along with 95% Confidence Intervals (CI) facilitated the quantification of the magnitude and directionality of the observed associations. Furthermore, subgroup analyses were conducted to scrutinize the variability in the prevalence and intensity of vertigo and auditory impairments, contingent upon demographic and clinical parameters. A significance threshold was established at *P* < .05 for all statistical inferences.

## 3. Results

Table 1 meticulously delineates the demographic and clinical characteristics of the study participants, alongside their auditory evaluation results, providing a comprehensive portrait of the cohort. The study encompassed 250 participants, presenting a diverse demographic spectrum. The mean age was 45.6 years, with a standard deviation of 10.3 years, reflecting a broad age range within the group. The gender distribution was relatively balanced, with 130 males (52%) and 120 females (48%). Occupation-wise, the cohort was diverse, encompassing 50 healthcare professionals (20%), 90 office workers (36%), and 110 individuals from various other professions (44%). The study also considered comorbidities: hypertension was noted in 30 participants (12%), diabetes in 20 (8%), and respiratory diseases in 15 (6%). The distribution of COVID-19 severity was also diverse, with 150 participants (60%) experiencing mild cases, 70 (28%) moderate cases, and 30 (12%) severe cases, providing a nuanced perspective on the impact of the virus across different severity levels.

**Table 1:** Participant characteristics and hearing assessment results.

Characteristic	Frequency (%)
Total participants	250
Age (Mean ± SD)	45.6 ± 10.3
Gender (Male/female)	130 (52)/120 (48)
Occupation	
Healthcare	50 (20)
Office worker	90 (36)
Others	110 (44)
Comorbidities	
Hypertension	30 (12)
Diabetes	20 (8)
Respiratory disease	15 (6)
COVID-19 severity	
Mild	150 (60)
Moderate	70 (28)
Severe	30 (12)
**Hearing parameter**	**Mean ± SD (dB HL**)
Pure-Tone Audiometry (250 Hz)	10.5 ± 5.2
Pure-Tone Audiometry (1000 Hz)	12.8 ± 6.0
Pure-Tone Audiometry (4000 Hz)	20.2 ± 8.3
Speech Discrimination Score (SDS)	94.6 ± 3.7
Tympanometry (Type A/B/C)	85/10/5
Otoacoustic Emissions (Pass/Fail)	90/10

Frequency (%) indicates the number and percentage of participants falling into each category. Mean ± SD (dB HL): Mean value and standard deviation (SD) of the hearing parameter measured in dB HL, Pure-Tone Audiometry (250 Hz, 1000 Hz, 4000 Hz): Results of pure-tone audiometry testing at different frequencies. SDS: Result of speech discrimination testing, indicating the ability to discern speech, Tympanometry (Type A/B/C): Distribution of tympanogram types A, B, and C, representing middle ear function, Otoacoustic Emissions (Pass/Fail): Results of otoacoustic emissions testing, indicating cochlear function.

COVID-19 = coronavirus disease 2019, dB HL = decibel hearing level, SDS = speech discrimination score.

**Figure 1.**
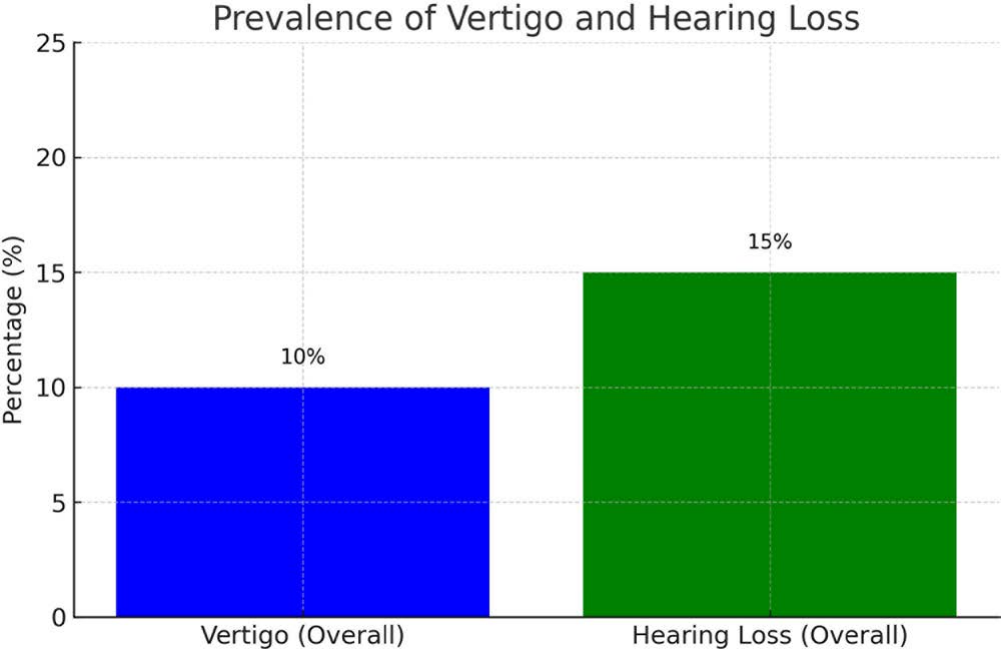
Prevalence of vertigo and hearing loss.

**Figure 2.**
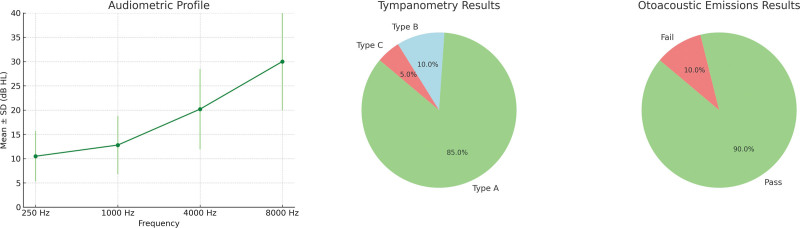
Comprehensive hearing assessment: audiometry trends and tympanometry distribution.

Figure 1 offers an incisive summary of vertigo prevalence and its varying degrees of severity among individuals post-COVID-19 recovery. The study identified vertigo as a common symptom, observed in 10% (25 participants) of the cohort. A detailed assessment of vertigo’s severity revealed that 6% (15 participants) experienced mild vertigo, 3.2% (8 participants) had moderate vertigo, and 0.8% (2 participants) experienced severe vertigo. Figure 2 showcases the results of auditory assessments conducted within the group. Utilizing Pure-Tone Audiometry at multiple frequencies, the study revealed an average hearing level of 10.5 dB HL (±5.2) at 250 Hz, 12.8 dB HL (±6.0) at 1000 Hz, and a marked increase to 20.2 dB HL (±8.3) at 4000 Hz. This escalating hearing level at higher frequencies suggests a potential decline in hearing sensitivity in this range. SDS averaged 94.6 (±3.7), indicating a generally robust speech comprehension capability among the participants. Tympanometry outcomes revealed that 85% of the cohort had Type A tympanograms, indicative of normal middle ear function. Conversely, 10% exhibited Type B, and 5% Type C tympanograms, pointing to potential middle ear anomalies. Lastly, Otoacoustic Emissions testing, a measure of cochlear health, demonstrated that 90% of participants exhibited satisfactory cochlear function, with only 10% failing the test.

**Figure 3.**
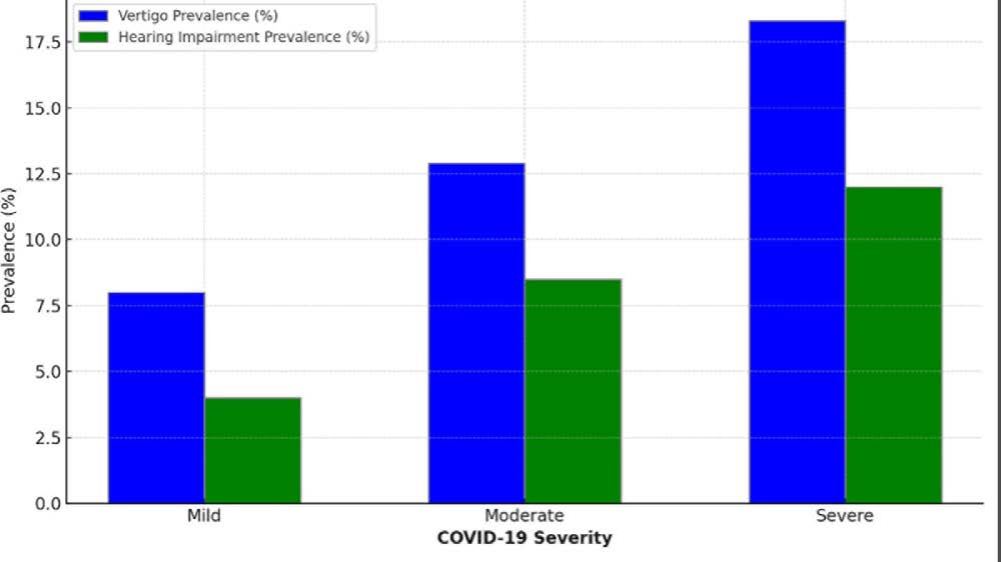
Comparative analysis of vertigo and hearing impairment prevalence across COVID-19 severity levels.

A comparative overview of the prevalence of vertigo and hearing impairment across different levels of COVID-19 severity is shown in Figure 3. The analysis revealed a clear pattern correlating the severity of COVID-19 with an increase in the prevalence of both vertigo and hearing impairment. In cases classified as mild COVID-19, the prevalence of vertigo was 8.0%, and hearing impairment was 4.0%. As the severity of COVID-19 escalated to moderate, the prevalence of vertigo increased to 12.9%, and hearing impairment rose to 8.5%. Notably, in severe COVID-19 cases, the prevalence of vertigo peaked at 18.3%, and hearing impairment was observed in 12.0% of the cases.

**Figure 4.**
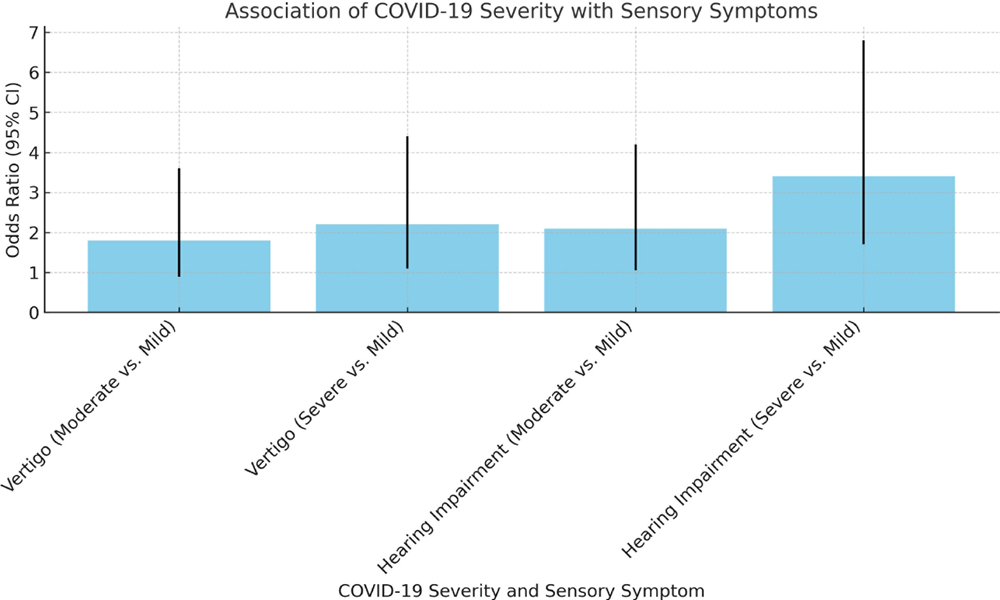
Odds ratios for vertigo and hearing impairment across COVID-19 severity levels.

The results from Table 2 and Figure 4, based on logistic regression analysis, provide insights into the association between the severity of COVID-19 and the incidence of sensory symptoms, namely vertigo and hearing impairment. The analysis compares the odds ratios of these symptoms in moderate and severe COVID-19 cases against mild cases. For vertigo, in patients with moderate COVID-19, the odds of experiencing vertigo were 1.80 times higher compared to those with mild COVID-19, with a confidence interval (CI) of 0.90 to 3.60; however, this was not statistically significant (*P* value = .091). The likelihood of vertigo increased more substantially in severe cases, where the odds ratio was 2.20 (95% CI: 1.10–4.40), and this association was statistically significant (*P* value = .031). Regarding hearing impairment, the analysis showed that patients with moderate COVID-19 had 2.10 times higher odds of experiencing hearing impairment than those with mild COVID-19 (95% CI: 1.05–4.20), with a statistically significant *P* value of .019. The association was even stronger in severe COVID-19 cases, where the odds ratio rose to 3.40 (95% CI: 1.70–6.80), with a highly significant *P* value of .001.

**Table 2:** Logistic regression analysis.

COVID-19 severity comparison	Sensory symptom	Odds ratio (95% CI)	*P* value
Moderate vs Mild	Vertigo	1.80 (0.90–3.60)	.091
Severe vs Mild	Vertigo	2.20 (1.10–4.40)	.031
Moderate vs Mild	Hearing Impairment	2.10 (1.05–4.20)	.019
Severe vs Mild	Hearing Impairment	3.40 (1.70–6.80)	.001

CI = Confidence Interval, Odds Ratio (95% CI) = Odds ratio with corresponding 95% confidence interval (CI), indicating the strength of association between COVID-19 severity and the outcome.

## 4. Discussion

The findings of this study provide valuable insights into the potential associations between COVID-19 and sensory symptoms, specifically vertigo and hearing impairment, among individuals who have recovered from the infection. These results contribute to our understanding of the multifaceted nature of COVID-19 and the potential long-term consequences it may have on the auditory and vestibular systems.

The observed correlation between COVID-19 severity and increased prevalence of vertigo and hearing impairment raises questions about the underlying biological mechanisms. One hypothesis is the direct viral impact of SARS-CoV-2 on the inner ear structures or auditory pathways.^[22]^ Alternatively, the systemic inflammation and immune responses triggered by COVID-19 might indirectly contribute to these sensory impairments.^[12]^ This aspect aligns with existing literature suggesting that systemic viral infections can lead to inner ear inflammation, resulting in auditory and vestibular dysfunctions.^[12]^ The prevalence of vertigo among COVID-19 survivors in our study was 10%, with varying degrees of severity.^[31]^ Comparatively, our findings indicate a higher prevalence of vertigo among COVID-19 survivors than in the general population, resonating with emerging reports that suggest a potential link between COVID-19 and vertigo. However, our results deviate from studies that report more pronounced hearing impairments in COVID-19 survivors, emphasizing the need for further research in diverse cohorts to understand these discrepancies.

In terms of hearing impairment, our study revealed that COVID-19 survivors generally exhibited good hearing sensitivity, as indicated by mean pure-tone audiometry thresholds within the normal range. The high mean SDS of 94.6 suggests excellent speech understanding among participants.^[32]^ Additionally, most participants had Type A tympanograms, indicating normal middle ear function.^[7]^ These findings are reassuring and suggest that the majority of COVID-19 survivors in our cohort did not experience significant hearing loss or middle ear dysfunction.^[7]^

Our study’s analysis demonstrated a notable association between COVID-19 severity and the prevalence of both vertigo and hearing impairment. Participants with severe COVID-19 were more likely to experience severe vertigo and severe hearing impairment compared to those with mild COVID-19. This finding raises intriguing questions about the potential mechanisms through which COVID-19 may influence the auditory and vestibular systems.^[7]^ The observed association between severe COVID-19 and sensory symptoms is consistent with reports of the virus’s systemic effects.^[33,34]^ COVID-19 is known to elicit a robust immune response, which may extend to affect other organ systems, including the inner ear and vestibular system.^[12]^ Previous research has suggested that viral infections can lead to inner ear inflammation and subsequent vestibular symptoms.^[16]^ Therefore, it is plausible that the immune response triggered by severe COVID-19 contributes to vertigo and hearing impairment in these cases.^[16]^ Moreover, severe COVID-19 is often associated with systemic complications and prolonged hospitalizations, during which patients may be exposed to various medications and treatments, some of which could potentially impact auditory and vestibular function.^[35]^ Future research should explore the specific mechanisms underlying these associations, including the role of immune responses, viral invasion, and treatment-related factors.^[36]^

Understanding the potential link between COVID-19 severity and sensory symptoms has several clinical implications. First and foremost, healthcare providers should be vigilant in assessing and monitoring patients who have recovered from severe COVID-19 for auditory and vestibular symptoms.^[37]^ Early detection and management of these symptoms are crucial for improving patients’ quality of life and mitigating long-term consequences.^[38,39]^ Additionally, these findings emphasize the importance of comprehensive rehabilitation and support for individuals who experience sensory symptoms following COVID-19 recovery.^[39]^ Audiological and vestibular assessments should be integrated into post-COVID care to identify and address any deficits promptly.^[40]^ From a public health perspective, raising awareness about the potential long-term effects of COVID-19, including sensory symptoms, is crucial.^[41]^ Healthcare systems need to be prepared for potential increases in consultations related to sensory symptoms in post-COVID patients.^[42]^ Additionally, these findings underscore the importance of comprehensive rehabilitation and support for individuals experiencing post-recovery sensory symptoms.^[43]^

While this study provides valuable insights, it is not without limitations. The cross-sectional design limits our ability to establish causality or determine the temporal relationship between COVID-19 severity and sensory symptoms. Longitudinal studies tracking individuals over time would be beneficial for elucidating the trajectory of these symptoms. Furthermore, our study did not explore the specific mechanisms underlying the observed associations. Future research should investigate the immunological, virological, and treatment-related factors contributing to vertigo and hearing impairment in COVID-19 survivors.

## 5. Conclusions

In conclusion, our study suggests a potential association between COVID-19 severity and the prevalence of vertigo and hearing impairment among individuals who have recovered from the infection. These findings underscore the importance of ongoing research to elucidate the mechanisms underlying these sensory symptoms and their long-term implications. Comprehensive post-COVID care that includes auditory and vestibular assessments is essential to address these symptoms and improve the overall well-being of COVID-19 survivors. Understanding the multifaceted impact of COVID-19 on various physiological systems, including the auditory and vestibular systems, is essential for providing holistic healthcare and support to individuals affected by this global health crisis.

## Author contributions

**Conceptualization:** Sarah Alshehri, Khalid A. Alahmari.

**Data curation:** Sarah Alshehri, Khalid A. Alahmari.

**Formal analysis:** Sarah Alshehri, Khalid A. Alahmari.

**Funding acquisition:** Sarah Alshehri.

**Investigation:** Sarah Alshehri, Khalid A. Alahmari.

**Methodology:** Sarah Alshehri, Khalid A. Alahmari.

**Project administration:** Sarah Alshehri.

**Resources:** Sarah Alshehri, Khalid A. Alahmari.

**Software:** Sarah Alshehri.

**Supervision:** Sarah Alshehri.

**Validation:** Sarah Alshehri.

**Visualization:** Sarah Alshehri.

**Writing – original draft:** Sarah Alshehri, Khalid A. Alahmari.

**Writing – review & editing:** Sarah Alshehri, Khalid A. Alahmari.
